# Update on the endorsement of CONSORT by high impact factor journals: a survey of journal “Instructions to Authors” in 2014

**DOI:** 10.1186/s13063-016-1408-z

**Published:** 2016-06-24

**Authors:** Larissa Shamseer, Sally Hopewell, Douglas G. Altman, David Moher, Kenneth F. Schulz

**Affiliations:** Centre for Practice-Changing Research, Ottawa Hospital Research Institute, Ottawa, ON K1H 8L6 Canada; School of Epidemiology, Public Health and Preventative Medicine, University of Ottawa, Ottawa, ON K1H 8M5 Canada; Centre for Statistics in Medicine, Nuffield Department of Orthopaedics, Rheumatology and Musculoskeletal Sciences, University of Oxford, Oxford, OX3 7LD UK; International Clinical Sciences Support Center, FHI 360, Durham, NC 27713 USA; Department of Obstetrics and Gynecology, UNC School of Medicine, Chapel Hill, NC 27599 USA

**Keywords:** CONSORT, Trial, Reporting, Endorsement, Registration

## Abstract

**Background:**

The CONsolidated Standards Of Reporting Trials (CONSORT) Statement provides a minimum standard set of items to be reported in published clinical trials; it has received widespread recognition within the biomedical publishing community. This research aims to provide an update on the endorsement of CONSORT by high impact medical journals.

**Methods:**

We performed a cross-sectional examination of the online “Instructions to Authors” of 168 high impact factor (2012) biomedical journals between July and December 2014. We assessed whether the text of the “Instructions to Authors” mentioned the CONSORT Statement and any CONSORT extensions, and we quantified the extent and nature of the journals’ endorsements of these. These data were described by frequencies. We also determined whether journals mentioned trial registration and the International Committee of Medical Journal Editors (ICMJE; other than in regards to trial registration) and whether either of these was associated with CONSORT endorsement (relative risk and 95 % confidence interval). We compared our findings to the two previous iterations of this survey (in 2003 and 2007). We also identified the publishers of the included journals.

**Results:**

Sixty-three percent (106/168) of the included journals mentioned CONSORT in their “Instructions to Authors.” Forty-four endorsers (42 %) explicitly stated that authors “must” use CONSORT to prepare their trial manuscript, 38 % required an accompanying completed CONSORT checklist as a condition of submission, and 39 % explicitly requested the inclusion of a flow diagram with the submission. CONSORT extensions were endorsed by very few journals. One hundred and thirty journals (77 %) mentioned ICMJE, and 106 (63 %) mentioned trial registration.

**Conclusions:**

The endorsement of CONSORT by high impact journals has increased over time; however, specific instructions on how CONSORT should be used by authors are inconsistent across journals and publishers. Publishers and journals should encourage authors to use CONSORT and set clear expectations for authors about compliance with CONSORT.

## Background

The CONsolidated Standards Of Reporting Trials (CONSORT) guideline was first published 20 years ago. Since that time, it has received widespread attention, including being lauded as a twentieth century milestone in health research methodology [1]. CONSORT is intended for use as a guide to reporting essential components of trial methods and findings by those preparing and reviewing reports of randomized trials.

The uptake of CONSORT is reflected in a number of metrics. Combined, the 1996 [2], 2001 [3], and 2010 [4] publications of the CONSORT Statement and Explanation and Elaboration documents [5, 6] have been cited more than 12,000 times (according to Scopus, May 2015), making CONSORT among the most highly cited biomedical publications of all time. Perhaps a more reflective measure of CONSORT uptake, however, is the support from a number of major editorial organizations (i.e., the International Committee of Medical Journal Editors, Committee on Publication Ethics, and World Association of Medical Editors) and its endorsement by more than 600 biomedical journals. Endorsement of CONSORT is typically demonstrated by a statement in a journal’s “Instructions to Authors” indicating support for CONSORT or a recommendation or requirement for authors to adhere to CONSORT when submitting a manuscript of a randomized trial for publication consideration.

A systematic review published in 2012 determined that journal endorsement of CONSORT was associated with more completely reported trials, based on assessments of more than 16,000 trials [7]. Four CONSORT checklist items (scientific rationale, sample size, sequence generation, and allocation concealment) and a summary score (including varying checklist items across studies reporting this) were significantly more likely to be reported in trials published in endorsing journals compared to their nonendorsing counterparts, and almost all remaining items were more completely reported, although this result was not statistically significant. However, reviews of publications also show that the reporting of key items remains well short of acceptable, with the reporting of details of random-allocation procedures being especially poor.

The extent to which journals are enforcing or checking for adherence to CONSORT in submitted/published trials is unknown and difficult to ascertain. To date, the best and most practical way to determine a journal’s stance on the matter of endorsement is to identify whether journals make recommendations around CONSORT in their “Instructions to Authors.” Two earlier studies have characterized CONSORT’s endorsement across high impact factor biomedical journals over time. In 2003, 22 % of high impact factor journals endorsed CONSORT [8]—this increased to 38 % in 2007 [9]; however both studies concluded that many journals used ambiguous language in terms of what was meant by endorsement.

This study provides an up-to-date assessment of the extent and nature of CONSORT endorsement in high impact factor journals in 2014 (the first since publication of CONSORT 2010) and describes changes in endorsement over time.

## Methods

### Selection of included journals

Journals were selected using the strategy adopted in the previous studies [8, 9]. Briefly, this consisted of using 2012 impact factors (Thomson Reuters Journal Citation Reports-Science Citation Index Expanded) to select the top five impact factor journals for each of 33 medical specialties and the top 15 impact factor journals in general and internal medicine. Journals that either explicitly indicated or were found not to publish clinical trials, as determined after a PubMed search (or an inspection of the journal scope when unsure), were excluded and replaced by the next one on the list. Journals that appeared in more than one specialty were not replaced by another journal but were included only once in the analysis.

### Survey of the “Instructions to Authors” published by the journals

One assessor (LS) examined the “Instructions to Authors” on the website of each included journal and extracted the information of interest for each journal between July and December 2014. Specifically, we extracted information on whether CONSORT and/or its extensions (for abstracts [10], acupuncture trials [11], cluster trials [12], harms outcomes [13], herbal interventions [14], noninferiority and equivalence trials [15], nonpharmacological interventions [16], patient-reported outcomes [17], and pragmatic trials [18]) were mentioned and, if so, assessed the following: whether CONSORT or any extension was mentioned as a “requirement” or as a “recommendation” or was unclear; which version of CONSORT (1996 [2], 2001 [3, 5], 2010 [4, 6]) was referenced, if any; and which CONSORT document (website, Statement paper, Explanatory paper, checklist, flow diagram, or other) was referred to, if any. If journals “required” CONSORT as a condition of submission, we extracted whether a flow diagram or checklist was an explicit requirement. While we aimed to examine endorsement of any official CONSORT extensions, we recognize that some types of trials for which CONSORT extensions exist (e.g., acupuncture or herbal interventions) may be published in niche journals not included in our sample [19]. In accordance with the original search strategy for this study, we made no specific attempt to identify/include such journals.

Any mention of ICMJE and reference to clinical trial registration (“recommended” or “required”) were also sought and extracted. Identification of the publisher of the included journals was also sought.

### Analysis

Data were summarized descriptively using frequencies. We examined whether any mention of ICMJE and trial registration was associated with CONSORT endorsement (relative risk and 95 % confidence interval) and summarized the journal endorsement status for publishers with more than one journal included in our sample. We present our data together with findings from previous iterations of this study [8, 9], and we compared the stability of the endorsement status for journals that appeared in more than one study year.

## Results

One hundred and eighty journals were identified in Thomson Reuters’ Journal Citation Reports database, of which, 12 were duplicates; thus, 168 journals were included in our sample.

### CONSORT endorsement

Of the 168 journals, 106 (63 %) mentioned CONSORT in their online “Instructions to Authors,” compared to 38 % (62/165) in 2007 and 22 % (36/166) in 2003. This is a relative increase of 66 % since 2007 and 186 % since 2003. Eighty-nine journals were included in all 3 years of the study; of these, 26 (29 %) became endorsers between 2003 and 2007, and 18 (20 %) endorsed CONSORT sometime between 2007 and 2014. No journals reversed their endorsement across the study years.

While 44 journals (42 %) *required* authors to use CONSORT, and 56 journals (53 %) *recommended* its use, fewer journals stated that submission of a completed checklist (*n* = 38) or a flow diagram (*n* = 39) was required as a condition of submission (Table 1). The 44 journals requiring authors to use CONSORT used explicit language, e.g. “Authors of trials must adhere to the CONSORT reporting guidelines appropriate to their trial design,” or “before the trial can undergo peer review, authors must provide a completed CONSORT checklist as a supporting file.” Journals recommending CONSORT (*n* = 56) used less forceful language, e.g. “Authors should adhere to these guidelines when drafting their manuscript.” In six journals, CONSORT was mentioned, but the extent of the journal’s recommendation was unclear, e.g., “Editorial assistance includes, but is not limited to, providing specific guidance regarding transparent reporting of items mentioned in pertinent reporting standards (e.g., CONSORT, PRISMA).” Seventy-three journals (69 %) referred to the CONSORT checklist, and 57 (54 %) referred to the flow diagram. In addition, while we did not set out to collect this information, we noted that several journals informed authors of how the checklist would be used during the peer review process; for example, “peer reviewers will be asked to refer to these checklists when evaluating such studies.”

**Table 1:** Mentions of CONSORT, ICMJE, and trial registration in the “Instructions to Authors” from the journals^1^

		2014	2007 ^2^	2003^3^
		(2012 IF)	(2006 IF)	(2001 IF)
		*N* = 168 (%)	*N* = 165 (%)	*N* = 166 (%)
**CONSORT Statement**		**106 (63 %)**	**62 (38 %)**	**36 (22 %)**
	Required	44 (42 %)	23 (37 %)	8^a^
	Recommended	56 (53 %)	39 (63 %)	26
	Can’t tell^*^	6 (6 %)	-	2
	Submit with checklist	38 (36 %)	17 (27 %)	nc
	Submit with flow diagram	39 (37 %)	nc	nc
Web address		84 (79 %)	46^b^ (58 %)	15 + 1^c^ (44 %)
Checklist		73 (69 %)	nc	nc
Flow diagram		57 (54 %)	nc	nc
2010 Statement		18 (17 %)	nr	nr
2010 E&E		0 (0 %)	nr	nr
2001 Statement		11 (10 %)	18 (29 %)	16 (44 %)
2001 E&E		0 (0 %)	1 (2 %)	3 (8 %)
1996 Statement		2 (2 %)	6^d^ (10 %)	9^d^ (17 %)
No specific reference/document indicated		5 (5 %)	0 (0 %)	0 (0 %)
**ICMJE**		**130 (77 %)**	**69 (42 %)**	**72 (43 %)**
Web address		117 (90 %)	48 (70 %)	23 (40 %)
Up-to-date reference^**^		41 (32 %)	3 (4 %)	27 (38 %)
Obsolete reference		59 (45 %)	15 (22 %)	41 (57 %)
Other		4 (3 %)	nc	nc
No specific reference		3 (2 %)	6 (9 %)	4 (6 %)
**Trial registration**		**106 (63 %)**	**61 (37 %)**	**nc**
	Required	78 (74 %)	44 (72 %)	nc
	Recommended	25 (24 %)	17 (28 %)	-
	Can’t tell	3 (3 %)	-	-
ICMJE (only)		27 (25 %)	23 (38 %)	nc
ClinicalTrials.gov (only)		9 (8 %)	9 (15 %)	nc
WHO International Clinical Trial Portal (only)		12 (11 %)	4 (7 %)	nc
Cites combination of the above		41 (39 %)	10 (16 %)	-
Other registry		3 (3 %)	-	-
None		14 (13 %)	-	-

*IF* impact factor, *CONSORT* Consolidated Standards of Reporting Trials, *nc* not collected, *nr* not relevant, *E&E* Explanation and Elaboration, *ICMJE* International Committee of Medical Journal Editors, *WHO* World Health Organization

^1^Bolded rows are the denominator for the numbers immediately following

^2^Data from Hopewell *et al.* [9]

^3^Data from Altman [8]

^a^Collapsed from Altman [8]: Required: required (3), must (5). Recommended: should (18), strongly encouraged (1), encouraged (2), recommended (2), please (2), may wish to consider (1). Can’t tell: see (1) and no directive comment (1).

^b^Web address was misspelled (*n* = 2)

^c^Reference to JAMA website

^d^Reference to an article citing the 1996 CONSORT Statement (*n* = 1)

^*^Language ambiguous

^**^For 2014, this was any clear reference to the “Recommendations for the conduct, reporting, editing, and publication of scholarly work in medical journals”; for 2007 and 2003 this was a > 2000 word article entitled “Uniform requirements for manuscripts submitted to biomedical journals: writing and editing for biomedical publication”

The CONSORT website, which launched in 2005, was mentioned in the “Instructions to Authors” in 46 (58 %) of the endorsing journals in 2007 compared to being mentioned in 84 (79 %) of the endorsers in 2014. Eighteen (17 %) of 106 endorsing journals referenced the most up-to-date version of CONSORT (CONSORT 2010), 11 (10 %) referenced the CONSORT 2001 Statement, and two (2 %) cited the original version of CONSORT (1996). Indeed, among journals mentioning CONSORT, 83 % did not reference the most current CONSORT Statement. No endorsing journals referred either to the 2001 or 2010 CONSORT Explanatory documents. Seventy-five (71 %) of the endorsing journals did not reference any CONSORT publication. Nine journals (8 %) referred to both the website and the CONSORT Statement. Five journals (5 %) did not refer to any specific CONSORT document or to the website.

### CONSORT extension endorsement

Only 22 of the 168 included journals (13 %) mentioned any of the nine CONSORT extensions published at the time of searching, of which, all except one also endorsed CONSORT (Table 2). No journals in our sample endorsed the CONSORT extension for acupuncture interventions [11]. Of note, the Abstracts and Harms extensions were explicitly incorporated into the CONSORT 2010 checklist.

**Table 2:** Mention of the CONSORT extensions published before December 2014 in “Instructions to Authors” on journal websites

	2014
	(2012 Impact factor)
	*N* = 168 (%)
Abstracts Extension (2008)	11 (7 %)
Required	0
Recommended	10
Can’t tell	1
Submit with checklist	0
Acupuncture Extension (STRICTA) (2001, updated 2010)	0 (0 %)
Required	0
Recommended	0
Can’t tell	0
Submit with checklist	0
Cluster Trials Extension (2004, updated 2012)	11 (7 %)
Required	4
Recommended	4
Can’t tell	3
Submit with checklist	0
Harms Extension (2004)	9 (5 %)
Required	5
Recommended	1
Can’t tell	3
Submit with checklist	0
Herbal interventions Extension (2006)	2 (1 %)
Required	0
Recommended	1
Can’t tell	1
Submit with checklist	0
Noninferiority Extension (2006, updated 2012)	4(2 %)
Required	0
Recommended	3
Can’t tell	1
Submit with checklist	0
Nonpharmacological Extension (2008)	4 (2 %)
Required	1
Recommended	1
Can’t tell	2
Submit with checklist	1
Pragmatic Trials Extension (2008)	2 (1 %)
Required	0
Recommended	2
Can’t tell	0
Submit with checklist	0
Patient Reported Outcomes Extension (2013)	1 (0.6 %)
Required	0
Recommended	0
Can’t tell	1
Submit with checklist	0

### ICMJE and trial registration

One hundred thirty journals (77 %) mentioned ICMJE in their “Instructions to Authors” in 2014, a large increase from 42 % (*n* = 69/165) of the journals in 2007 and 43 % (*n* = 72/166) in 2003 (Table 1). One hundred seventeen (90 %) provided a link to the ICMJE website, 40 (34 %) of which also mentioned the most recent ICMJE guidance [20]. While 59 (50 %) cited an older version of the ICMJE recommendations, most (92 %) also provided a link to the ICMJE website where the most up- to-date documents are hosted. Four further journals (3 %) referenced specific ICMJE recommendations (e.g., “ICMJE criteria for authorship”) but provided no citation or link to an ICMJE document. Three journals (2 %) mentioned ICMJE but did not provide any link or reference. Journals that referred to CONSORT were more likely to refer to ICMJE in the “Instructions to Authors” (97/106, 92 %) than those journals not referring to CONSORT (33/62, 53 %) (relative risk 1.72, 95 % confidence interval [CI] 1.35 to 2.19).

Of the 168 journals in the sample, 106 (63 %) mentioned trial registration (CONSORT 2010 checklist item 23) in their “Instructions to Authors” (Table 1); 78 (74 %) *required* registration of submitted trials, 25 (24 %) *recommended* registration, and three made no specific statement of support for registration. Furthermore, 27/106 journals (35 %) referred only to the ICMJE statement about trial registration (which references at least seven registry options) [21], 9/106 (8 %) referred only to clinicaltrials.gov, 12/106 (11 %) referred only to the World Health Organization Clinical Trials Platform [22], and 41/106 (39 %) journals referred to a combination of these three. Thirteen journals (12 %) mentioned trial registration but did not specifically mention or provide a link to one or more trial registries. Journals mentioning CONSORT were more likely to mention trial registration (91/106, 86 %) than those not mentioning CONSORT (15/62, 24 %) (relative risk 3.55; 95 % CI 2.27 to 5.55).

### CONSORT endorsement across publishers of high impact journals

The 168 included journals were published by 39 publishers, 14 of whom published more than one of the journals. The ratio of endorsing journals to nonendorsing journals was inconsistent across journals (Table 3). In our sample, only one publisher with more than five journals included in the sample (the American Medical Association) had all endorsing journals and no nonendorsing journals.

**Table 3:** CONSORT endorsement status by publishers with more than one journal included in the sample

Publisher Name	# of journals included in sample	Endorsers:Nonendorsers
AMERICAN DIABETES ASSOCIATION	2	1:1
AMERICAN MEDICAL ASSOCIATION	6	6:0
AMERICAN PHYSIOLOGICAL SOCIETY	2	0:2
AMERICAN SOCIETY OF NEPRHOLOGY	2	2:0
AMERICAN SOCIETY OF NUTRITION	2	2:0
BIOMED CENTRAL	4	3:1
BMJ PUBLISHING GROUP	5	4:1
ELSEVIER	43	28:15
LIPPINCOTT WILLIAMS & WILKINS	20	15:5
MARY ANN LIEBERT	2	0:2
NATURE PUBLISHING GROUP	10	6:4
OXFORD UNIVERSITY PRESS	10	3:7
PUBLIC LIBRARY OF SCIENCE	3	3:0
SAGE PUBLICATIONS	2	2:0
SPRINGER	5	1:4
WILEY-BLACKWELL	27	15:12

## Discussion

The CONSORT Statement continues to gain traction among the biomedical journal community. As of December 2014, CONSORT was endorsed by 63 % of high impact journals – almost triple the number of endorsers since the first investigation of this kind was conducted in 2003 and a 63 % (relative) increase since 2007. While less than 20 % of the endorsing journals referenced the most up-to-date CONSORT Statement and none referred to either of the CONSORT Explanation and Elaboration papers, most (90 %) provide a link to the CONSORT website, which always provides access to the most recent CONSORT documents. Recently, we have seen that journal endorsement of CONSORT is associated with more completely reported trials compared to nonendorsement, based on a 2012 systematic review including over 16,000 trials [7].

These numbers are encouraging. However, while CONSORT is a set of standard reporting recommendations, its implementation is far from standardized. For example, many of the endorsing journals in our sample did not make strong statements about CONSORT; approximately one half of the journals stop short of *insisting* that authors follow the guideline. Approximately one quarter of the journals ask that both a checklist and flow diagram are included with the trial submission, one third of the journals ask authors to submit either one or the other, and approximately one half of the endorsing journals do not specifically ask or require authors to submit either a CONSORT checklist or flow diagram. Even among trials published in endorsing journals in the aforementioned review, the reporting of many CONSORT items is still poor [7]. Undoubtedly, authors may be unaware or confused, about journal expectations regarding author use and adherence to CONSORT. This is in agreement with findings from the aforementioned review, which indicated that seemingly large relative effects of endorsement did not always translate into similar absolute effects [7]. For instance, the review found that the description of allocation concealment is complete in 81 % more trials published in endorsing journals than in nonendorsing journals [7]. In absolute terms, this equated to only 45 % of the trials in the endorsing journals (*n* = 876) and 22 % of the trials published in the nonendorsing journals (*n* = 1,520). Given that allocation concealment is essential to maintaining trial validity, the fact that it is incompletely described in more than one half of trials is a serious concern. Therefore, a simple statement in a journal’s “Instructions to Authors” about using or adhering to CONSORT is likely not sufficient on its own for improving reporting to the extent needed for some checklist items.

Authors can and should do better. Likewise, peer reviewers need to recognize the importance of identifying deficiencies in reporting. A 2014 series in *The Lancet* called for increased value and reduced waste in research. The authors called on gatekeepers of the publishing and dissemination process, including journals, to help authors increase their awareness, capacity, and capabilities around optimal reporting practices [23, 24]. Journals should be assured that their efforts toward CONSORT endorsement leading to better trial reporting are worthwhile. However, a supporting statement in a journal’s “Instructions to Authors” is proving insufficient to improve trial reporting on a large scale, and more must be done. One study compared the reporting of trial abstracts in journals with varying levels of endorsement of the CONSORT for abstracts extensions [25]. It found that trials published in journals with a policy to actively implement the guideline (e.g., to send an email to authors to revise the abstract according to the guideline) were more completely reported than those in journals with no active policy [25].

In Box 1, we offer some unambiguous standard language that can be used by journals in their “Instructions to Authors” to demonstrate their support for CONSORT and indicate their expectations of the authors.

Collectively, the biomedical publishing industry is an important gatekeeper, determining which research reaches healthcare professionals and, ultimately, which is incorporated into patient-care decisions. The endorsement status of journals across publishers (Table 3) suggests that journals with the same publisher do not necessarily share a common set of “Instructions to Authors.” A standard set of “Instructions to Authors” across publishers, allowing tailored information for each journal as necessary, may help to ensure better author understanding of journal expectations (including those around reporting) and facilitate an efficient peer review process by providing consistent messaging about journal expectations. Doing so may also benefit publishers. In this industry, the inexorable pursuit of impact metrics continues. Publishing research that adheres to minimum reporting standards, such as those outlined in CONSORT, will produce more usable reports. For publishers, increasing the usability of a report may increase its ability to be included, and therefore cited, in future research.

Lastly, our survey sampled journals with the highest 2012 impact factors in the medical specialties and in general medicine. Almost all of the general medical journals included in our sample (13/15) endorse CONSORT, and most have been involved in efforts to improve research reporting quality and completeness over the past two decades. Some specialties/subspecialties are now taking on the responsibility of improving research reporting within their specialty using a top-down approach. For example, a 2014 editorial in the *Archives of Physical Medicine and Rehabilitation* outlining a commitment to enforce mandatory compliance with reporting guidelines, including CONSORT, has been co-published by at least 35 journals, mostly within the rehabilitation specialty. This creates a complete “circle” of endorsing journals within a subspecialty, so authors must comply if they want to publish their research in a relevant journal [26]. This top-down model within a subspecialty is being repeated elsewhere [27, 28].

## Conclusions

Many journals now endorse the CONSORT Statement, and such endorsement has been found to be associated with more complete reporting. Publishers and journals should encourage authors to use CONSORT and set clear expectations for authors about compliance with CONSORT (and indeed other reporting guidelines). They should make such information transparent and unambiguous in their “Instructions to Authors.” Doing so will help authors to prepare high-quality reports of randomized trials, leading to better quality evidence to inform the treatment and prevention of disease.

## Abbreviations

CONSORT, Consolidated Standards of Reporting Trials; ICMJE, International Committee of Medical Journal Editors

## Box 1. Recommended endorsement text for journals to include in their “Instructions to Authors”

“[Journal name] requires a completed CONSORT 2010 checklist and flow diagram as a condition of submission when reporting the results of a randomized trial. Templates for these can be found here or on the CONSORT website [www.consort-statement.org], which also describes several CONSORT checklist extensions for different designs and types of data beyond two-group parallel trials. At minimum, your article should report the content addressed by each item on the checklist. Meeting these basic reporting requirements will greatly improve the value of your trial report, may facilitate and/or enhance the peer review process, and may enhance the chance for eventual publication of your report."
